# Knowledge of Complementary Medicine and Therapies Among Family Physicians and the General Population in Saudi Arabia

**DOI:** 10.3390/healthcare14131930

**Published:** 2026-07-01

**Authors:** Safaa M. Alsanosi

**Affiliations:** Department of Pharmacology and Toxicology, Faculty of Medicine, Umm Al Qura University, Makkah 24382, Saudi Arabia; smsanosi@uqu.edu.sa

**Keywords:** complementary medicine, family physicians, Saudi Arabia

## Abstract

**Highlights:**

**What are the main findings?**

- Family physicians exhibited significantly greater knowledge of CMT compared to the general population. However, both groups demonstrated limited awareness of regulatory and quality-control measures.
- Healthcare professionals served as the primary source of CMT information for both physicians and the public. Significant differences were observed between these groups in terms of knowledge levels and information sources (*p* < 0.001).

**What are the implications of the main findings?**

- Public education and awareness initiatives are necessary to enhance general knowledge and promote the safe use of CMTs, particularly with respect to evidence-based practices and regulatory standards.
- Enhancing physician training in CMT regulation, safety, and quality assurance could improve the reliability of information provided to the public and facilitate safer integration of CMTs into healthcare practice.

**Abstract:**

**Background:** Complementary Medicine and Therapies (CMTs) are increasingly used worldwide, particularly in Saudi Arabia, due to growing interest in holistic and integrative healthcare. However, concerns regarding safety, regulatory awareness and evidence-based use persist, while comparative knowledge data between family physicians and the general population remain limited. This study aimed to compare knowledge of CMTs between family physicians and the general population in Saudi Arabia. **Methods:** A comparative cross-sectional analysis was conducted using two independent datasets collected in Saudi Arabia from a total of 1307 participants. Variables related to the participants’ demographic characteristics, and their knowledge of and information sources on CMTs, were harmonised prior to analysis. Between-group comparisons were performed using Chi-square and Fisher’s exact tests, with statistical significance set at *p* < 0.05. **Results:** The family physicians demonstrated significantly higher levels of knowledge of CMTs than the general population. While 35.5% of the physicians demonstrated good knowledge levels, 82.0% of the public participants exhibited poor or low knowledge. However, knowledge of regulatory and quality control measures governing CMTs remained limited among both the physicians (21.0%) and the public (24.6%). Both the physicians (66.1%) and the members of the public (34.8%) primarily relied on healthcare professionals as major sources of information on CMTs. Significant associations were observed between the participant group and the knowledge-related variables, overall knowledge levels and information sources (*p* < 0.001). **Conclusions:** Strengthening public education, physician training and regulatory awareness may support safer and more evidence-based integration of CMTs within healthcare systems.

## 1. Introduction

Over recent decades, the global utilisation of complementary medicine and therapies (CMTs) has expanded considerably, reflecting growing public interest in natural health products, preventive healthcare strategies and integrative therapeutic approaches [1]. CMTs encompass a broad range of healthcare practices and products that fall outside conventional biomedical treatment, including herbal medicines, nutritional supplements, traditional healing methods and mind–body interventions [2,3].

In many regions of the world, particularly across the Middle East and Asia, CMTs are frequently used alongside conventional medical treatments [4]. Their widespread use has been attributed to factors such as accessibility, affordability, cultural traditions and the perception that natural remedies are safer and more holistic than pharmaceutical therapies [5]. Previous studies have shown that many individuals utilise CMTs for chronic disease management, symptom relief, health promotion and disease prevention [5,6]. However, the growing popularity of CMTs has also raised important concerns regarding their safety, regulatory oversight and the adequacy of public knowledge surrounding their appropriate use [1].

Recent evidence has indicated that the use of CMTs continues to increase globally as individuals seek more personalised, holistic and patient-centred approaches to healthcare. In Saudi Arabia, herbal medicines, dietary supplements, traditional remedies and other complementary therapies remain widely used across diverse population groups [7]. Although these therapies are commonly perceived as beneficial and safe, concerns persist regarding inappropriate self-medication, limited awareness of potential herb–drug interactions, variability in product quality and the spread of inaccurate or non-evidence-based health information through social media and informal networks [8]. Such challenges highlight the growing need to ensure that healthcare professionals and the general public possess adequate knowledge of the benefits, risks, safety considerations and regulatory frameworks governing the use of CMTs [9].

In Saudi Arabia, CMTs are widely used by the general population. Herbal medicines and traditional remedies remain deeply rooted within cultural and healthcare practices, and they are commonly used either independently or in combination with conventional medical treatment [10]. Despite their widespread popularity, concerns persist regarding public awareness of potential risks associated with CMT use, including herb–drug interactions, variability in product quality, delayed medical care and the limited regulation of certain CMTs [11,12]. Insufficient knowledge of evidence-based indications, contraindications and safety considerations may increase the risk of inappropriate use and adverse health outcomes [13].

Healthcare professionals, particularly family physicians, play a pivotal role in promoting the safe and evidence-based integration of CMTs within healthcare systems [14]. As primary care providers and the first points of contact for many patients, family physicians are expected to possess adequate knowledge of commonly used CMTs, including their therapeutic efficacy, safety profiles and potential interactions with conventional medications [15]. However, previous research has demonstrated considerable variability in healthcare professionals’ knowledge, training and attitudes towards CMTs, which may influence their confidence and ability to counsel patients effectively [16].

Given this variability, understanding differences in the knowledge of and information sources on CMTs between healthcare professionals and the general population is of considerable importance. Investigating these differences may provide valuable insights into how information on CMTs is accessed, interpreted and utilised in community and clinical settings [17]. Knowledge gaps may influence patient decision-making, patterns of healthcare utilisation and the safe integration of CMTs into clinical practice [18]. Furthermore, inadequate communication between physicians and patients regarding CMT use may contribute to underreporting of such practices, thereby increasing the risk of adverse interactions, medication-related complications or delays in seeking appropriate conventional treatment [19].

The increasing use of CMTs has highlighted important regulatory and educational challenges [20]. Despite existing regulatory frameworks in Saudi Arabia, gaps remain in public awareness regarding product regulation, quality assurance and evidence-based use. Healthcare professionals frequently encounter patients using CMTs alongside conventional treatments, yet variations in physician knowledge and training persist [21]. Strengthening awareness and education among healthcare professionals and the public is essential to promote the safe and informed use of CMTs [20].

Due to the widespread use of CMTs in Saudi Arabia, several studies have explored knowledge and information about complementary and alternative medicine within specific populations, but comparative analyses between healthcare professionals and the public remain limited, particularly within the Saudi Arabian context [11,13,22,23]. This comparative cross-sectional secondary analysis was undertaken to assess and compare knowledge of CMTs, regulatory awareness and information sources among family physicians and the general population in Saudi Arabia. The study aimed to identify gaps in knowledge and awareness that may be addressed through targeted educational interventions and policy measures to support the safe and evidence-based use of CMTs.

## 2. Materials and Methods

### 2.1. Ethical Approval

The comparative-analytical approach utilised data from two cross-sectional surveys conducted in Saudi Arabia. Both studies received approval from the Biomedical Research Ethics Committee, Faculty of Medicine, Umm Al-Qura University, Makkah, Saudi Arabia, and adhered to the principles of the Declaration of Helsinki. All participants were informed of the study objectives, and participation was voluntary. The first dataset consisted of family physicians (Approval No. HAPO-02-K-012-2024-032080—31 March 2024), while the second dataset included members of the general public (Approval No. HAPO-02-K-012-2025-02-2550—16 February 2025).

### 2.2. Data Sources and Setting

A comparative cross-sectional secondary data analysis design was employed. Two independently collected survey datasets were analysed: one comprising family physicians practicing in Saudi Arabia (N = 62) and the other comprising members of the general public in Saudi Arabia (N = 1245). This design was appropriate for comparing knowledge of CMTs, awareness of CMT-related regulations and quality-control measures and sources of CMT-related information between two distinct population groups at a single point in time. Utilizing secondary datasets enabled the efficient examination of differences in knowledge and awareness within the same national context.

Given the cross-sectional nature of the study, the analysis can identify associations and differences between groups but cannot establish causal relationships. Therefore, although variations in knowledge levels were observed between family physicians and members of the general population, the study cannot determine whether professional training, clinical experience, educational attainment or other factors directly caused these differences.

To compare the knowledge of and information sources on CMTs between family physicians and the general population, a comparative analysis design was employed using data from two cross-sectional surveys conducted in Saudi Arabia. The physician dataset included family physicians practising in Saudi Arabia with different levels of clinical experience, including general practitioners, residents, specialists and consultants. The public dataset comprised adults spanning a range of age groups, educational backgrounds and socioeconomic levels.

### 2.3. Compliance with Strengthening the Reporting of Observational Studies in Epidemiology (STROBE)

This study was conducted and reported in accordance with the strengthening the reporting of observational studies in epidemiology (STROBE) statement for cross-sectional studies. The methodology, participant selection, variable harmonisation, statistical analyses and reporting of findings were designed to ensure methodological transparency, analytical consistency and comprehensive reporting of observational research. The discussion and interpretation of findings also considered potential sources of bias, study limitations and generalisability, in line with STROBE recommendations, as shown in Supplementary Table S1.

### 2.4. Sampling Strategy and Eligibility Criteria

The two datasets included in this comparative analysis were derived from independently conducted cross-sectional surveys in Saudi Arabia. Both questionnaires were developed based on previously published studies and adapted to meet the objectives of the original investigations, as shown in Supplementary Tables S2 and S3. The instruments were reviewed by subject-matter experts and bilingual Arabic–English professionals to ensure content validity, linguistic accuracy and cultural appropriateness. Both surveys were pilot tested prior to implementation, and minor revisions were made based on participant feedback. Participants were recruited using nonprobability sampling methods. The family physician dataset and the public dataset were recruited through online convenience sampling using electronic survey distribution platforms. The survey was distributed through social media and digital channels targeting all major regions. A total of 1245 completed questionnaires were analysed. The total number of individuals reached and the response rates are unknown due to the open online distribution.

Family Physician Dataset: Eligible participants were physicians practising in Saudi Arabia during the study period, including general practitioners, residents, specialists and consultants. The participants were required to be actively involved in clinical practice and to provide complete questionnaire responses. Incomplete responses were excluded from the final analysis. Public Dataset: Eligible participants were adults aged 18 years or older residing in Saudi Arabia during the study period. The participants were required to provide informed consent and complete the survey questionnaire. Incomplete responses were excluded from the final analysis. For the present secondary analysis, only eligible participants with complete responses and harmonisable variables relevant to the study objectives were included. Variables that could not be meaningfully harmonised between the two datasets were excluded from the comparative analyses.

### 2.5. Participant Flow and Final Analytical Sample

The present study utilised two independently collected cross-sectional survey datasets conducted in Saudi Arabia. The physician dataset contributed 62 eligible physician responses, while the public dataset contributed 1245 eligible responses from members of the general population. Only complete and eligible responses available within the original datasets were included in the comparative analysis.

Because both surveys were distributed through open online recruitment channels, information regarding the number of individuals who received survey invitations, initiated the survey, submitted incomplete questionnaires or were excluded because of duplicate responses was not available. Consequently, detailed participant flow metrics and response rates could not be determined. The final comparative analysis therefore included 1307 participants, comprising 62 physicians and 1245 members of the general population.

### 2.6. Data Harmonisation Procedures

Prior to comparative analysis, variables from the two independent datasets were systematically harmonised to ensure conceptual and analytical comparability, as shown in Supplementary Table S4. Variables assessing the participants’ demographic characteristics, knowledge of CMTs and related regulations, and CMT information sources were reviewed and mapped into standardised categories. Minor differences in questionnaire wording and response structures were reconciled by aligning categories and consolidating comparable responses into unified analytical variables.

Data harmonisation was undertaken to enable valid comparisons between two independently collected datasets that differed in questionnaire structure, wording and response formats. Variables relating to demographic characteristics, knowledge of CMTs, regulatory and quality-control awareness and information sources were systematically reviewed and aligned according to their conceptual meaning. Where differences in response categories existed, the responses were recoded into standardised categories to improve comparability. Only variables assessing comparable constructs across both datasets were retained, while items that could not be meaningfully aligned were excluded from the analysis. The resulting harmonised variables were subsequently used for descriptive and comparative analyses, ensuring analytical consistency between family physicians and members of the general population [24].

### 2.7. Knowledge Assessment and Scoring

Knowledge of CMTs was assessed using harmonised variables available in both datasets. The assessment encompassed the participants’ understanding of CMT concepts, awareness of regulatory and quality-control frameworks, safety considerations and evidence-informed use of CMTs. Overall CMT knowledge was evaluated using variables that assessed conceptual understanding, awareness of regulatory and quality-control measures and related knowledge domains [25,26]. Information source variables were analysed separately, as they represent channels of information acquisition rather than factual knowledge or awareness. Knowledge levels were categorised as poor (<30%), average (30–70%) and good (>70%) using percentage-based approaches commonly applied in exploratory cross-sectional knowledge assessment studies. Variables were harmonised across both datasets before analysis to ensure comparability and consistency with the study objectives.

### 2.8. Statistical Analysis

The primary construct examined was knowledge of CMT, defined as participants’ understanding of CMT concepts, regulatory and quality-control measures, safety considerations, evidence-based use and potential risks. Conceptual understanding referred to familiarity with the general principles of CMTs, while regulatory awareness reflected knowledge of the standards and oversight governing their use. Safety knowledge encompassed awareness of potential benefits, risks, adverse effects, contraindications and herb–drug interactions. Information sources were assessed separately and referred to the main channels through which participants obtained CMT-related information about, for example, healthcare professionals, scientific resources, social media and personal networks. These constructs were harmonised across both datasets to ensure conceptual consistency and facilitate valid comparisons between family physicians and members of the general population.

Descriptive statistics were used to summarise demographic characteristics and knowledge variables. Categorical variables are presented as frequencies and percentages. Overall CMT knowledge was evaluated by scoring the participants’ responses to structured knowledge-related questions on CMTs, including conceptual understanding, regulatory awareness, safety considerations and information sources. The public and physician groups were compared using the chi-square (χ^2^) test to examine the associations between the participant groups and the knowledge-related variables. When appropriate, Fisher’s exact test was applied to variables with small expected cell counts. A two-sided *p*-value of <0.05 was considered statistically significant. Statistical analyses were performed to evaluate differences in the knowledge of and information sources on CMTs between the two populations.

All demographic and knowledge-related variables were evaluated for missing data prior to the analysis. Only complete and eligible survey responses were included in the final datasets. For knowledge-related items, responses of ‘don’t know’ or ‘not sure’ were retained as valid, as they indicated the participants’ uncertainty or lack of awareness regarding the topic. Missing values are reported in descriptive analyses, where applicable. Participants with missing responses for the primary knowledge outcome were excluded from the overall knowledge score calculation using a complete case approach. Imputation procedures were not performed due to the minimal proportion of missing data. Sensitivity analyses were also not conducted [27,28].

## 3. Results

The demographic characteristics of the study population are summarised in Table 1. The physicians were predominantly (58.1%) aged 36–45 years, while most (56.2%) of the public participants were aged 18–29 years. Gender distribution also differed between groups: the physicians showed equal gender distribution (50.0% male and 50.0% female), whereas the public sample was predominantly (70.0%) female. Regarding education, in the physician group, most (66.1%) were consultants, whereas in the public group, 59.1% held a bachelor’s degree, while 28.3% had a high school education or less. Socioeconomic differences were likewise evident: two-thirds (66.1%) of the physicians reported monthly incomes exceeding 40,000 SAR, whereas over half of the public participants (53.3%) reported monthly incomes below 5000 SAR.

Table 2 summarises the comparative findings regarding knowledge of CMTs among the physicians and members of the public. With respect to basic knowledge of the CMT concept, 85.5% of the physicians and 72.6% of the public reported familiarity. Knowledge of regulatory and quality control measures governing CMTs was limited in both groups: 46.8% of the physicians were uncertain, whereas 42.8% of the public reported no awareness. Both the physicians (66.1%) and the members of the public (34.8%) primarily relied on healthcare professionals as major sources of information on CMTs.

**Figure 1:**
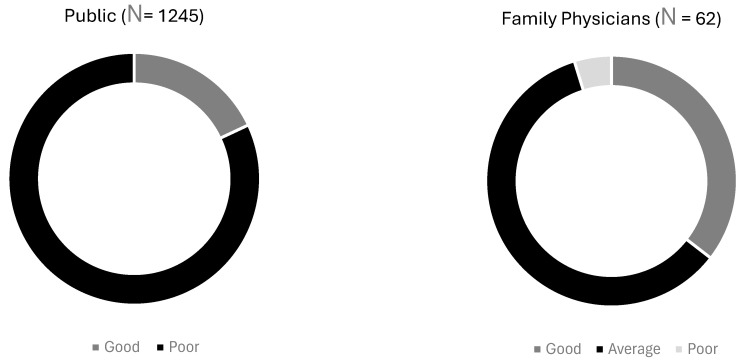
Overall CMT Knowledge among Family Physicians (N = 62) and Members of the Public (N = 1245) in Saudi Arabia.

As shown in Figure 1, marked differences were observed in the overall levels of CMT knowledge between the two groups. Among physicians, 59.7% demonstrated average knowledge, and 35.5% demonstrated good knowledge. In contrast, most public participants, 82.0%, demonstrated poor knowledge, while only 18.0% demonstrated good knowledge. To improve the interpretation of key study estimates, 95% confidence intervals (CIs) were calculated. Among family physicians, 35.5% demonstrated good knowledge of CMTs, corresponding to a 95% CI of 23.7–47.4%. In contrast, 82.0% of public participants demonstrated poor knowledge of CMTs, with a 95% CI of 79.9–84.1%. Awareness of regulatory and quality-control measures related to CMTs remained limited in both groups. Among family physicians, 21.0% reported awareness of existing regulations (95% CI: 10.8–31.1%), while 24.6% of members of the general population reported such awareness (95% CI: 22.2–27.0%). These confidence intervals indicate the precision of the estimated proportions and demonstrate the greater uncertainty surrounding estimates from the smaller physician sample compared with those from the substantially larger public sample.

The association between the participant groups and the knowledge-related variables is presented in Table 3. A statistically significant association was observed between the groups and basic CMT knowledge (*p* = 0.020), with the physicians demonstrating greater familiarity with CMT terminology than the public. Knowledge of CMT regulatory frameworks was borderline statistically significant (*p* = 0.050), reflecting moderate uncertainty about regulatory oversight in both groups. A significant association was also found between the participant groups and CMT information sources (*p* < 0.001). Both the physicians (and the members of the public (34.8%)) primarily relied on healthcare professionals as major sources of information on CMTs. Overall knowledge of CMTs differed significantly between the two groups (*p* < 0.001). The physicians demonstrated substantially higher knowledge levels than the public, highlighting a pronounced knowledge gap between healthcare providers and the population they serve.

## 4. Discussion

This comparative secondary analysis aimed to compare self-reported familiarity with CMTs, awareness of CMT-related regulatory and quality-control measures, sources of CMT-related information and exploratory CMT-related knowledge scores between family physicians and members of the general population in Saudi Arabia. The findings revealed a significant knowledge gap between healthcare providers and the general population, with important implications for public health education and clinical practice.

Because probability-based sampling methods were not employed, the findings should be interpreted as exploratory and may not be fully representative of all family physicians or members of the general population in Saudi Arabia. Consequently, caution is warranted when generalising the findings beyond the study sample. Participants were invited to participate through online survey distribution channels. Information regarding the number of individuals who received the survey invitation, accessed the survey link or declined participation was not available for either dataset. Consequently, response rates could not be calculated because the surveys were distributed through open online channels and the number of individuals who received the survey links was unknown [29]. Only completed and eligible responses were included in the final analyses. This limitation should be considered when interpreting the findings, as the potential for nonresponse bias cannot be excluded.

The smaller physician sample size compared with the public sample reflects the limited number of practising family physicians in Saudi Arabia. National reports have highlighted ongoing shortages in the family medicine workforce, with estimates of approximately 1400–1500 practising family physicians, despite substantially higher projected needs [30,31]. Several cross-sectional studies involving primary healthcare and family physicians in Saudi Arabia have used comparable physician sample sizes, particularly in exploratory and knowledge assessment designs [31,32]. Accordingly, the physician sample included in the present study was considered acceptable for comparative exploratory analysis. A key finding of this study was that family physicians demonstrated substantially higher levels of CMT knowledge than the public. Most of them had average to good knowledge, whereas most members of the public demonstrated poor knowledge. This disparity likely reflects the influence of formal medical training, professional exposure to clinical evidence and greater access to scientific literature among physicians [33]. In contrast, the public may have limited access to evidence-based information on CMTs [34,35].

Physicians and members of the public in this study relied primarily on healthcare professionals as their main sources of information regarding CMTs. This finding underscores the significant trust placed in healthcare providers in medical guidance and evidence-based recommendations. Previous research has also indicated that healthcare professionals remain among the most trusted sources of health-related information, even as the use of social media and online resources increases [36,37]. Members of the public often seek professional advice concerning the safety, efficacy and potential interactions of complementary therapies. Physicians may also depend on professional networks and colleagues for updated information about CMTs [38]. This reliance highlights the need to enhance healthcare professionals’ education and training in complementary medicine to ensure accurate patient counselling [37]. Expanding CMT-related education and professional development programs may facilitate safer and more evidence-based integration of complementary therapies into healthcare practice [39].

Although the physicians demonstrated higher overall knowledge levels, both groups showed limited knowledge of the regulatory frameworks governing CMTs. Only a minority of participants in either group reported familiarity with such knowledge. This gap, evident even among healthcare professionals, may have implications for patient counselling and the safe integration of CMTs into clinical practice [40]. The significant associations observed between the participant groups and the knowledge-related variables underscore the importance of strengthening education on CMTs. For healthcare professionals, these findings highlight the need for improved physician training and continuing professional education on CMTs to promote safer patient counselling and evidence-based integration of such therapies within healthcare systems [7]. For the public, improved access to reliable information on CMTs, particularly given the widespread use of herbal and traditional treatments in many communities, may support informed decision-making on the use of CMTs and reduce the risk of misinformation or unsafe practices [7,35].

Because the original datasets primarily assessed self-reported knowledge and awareness rather than objective factual knowledge, the findings should be interpreted as indicators of perceived knowledge and awareness. Responses indicating awareness of or familiarity with CMT-related concepts were used to characterise the participants’ knowledge levels. Responses of ‘Don’t know’ were classified as a lack of awareness regarding the respective topic. The test did not have predefined correct answers or an externally validated scoring key. Therefore, the resulting knowledge scores do not represent a direct measure of factual knowledge accuracy [12,41]. Instead, they provide a comparative assessment of the perceived knowledge and awareness of CMTs among family physicians and members of the general population. Overall scores were calculated based on the proportion of complete responses.

This study has several notable strengths. It represents one of the few comparative investigations of CMT-related knowledge, awareness and information sources among family physicians and the general population in Saudi Arabia. The inclusion of a large public sample broadens the scope of the analysis and enhances the robustness of the findings. Nevertheless, several limitations warrant consideration. The study relied on secondary analysis of two independently collected cross-sectional datasets, which restricted control over survey design, measurement and data collection. The physician sample was considerably smaller than the public sample, and demographic differences in age, education and income may have influenced the comparisons. Although a systematic harmonisation process was implemented to improve comparability, the integration of independently collected data may have resulted in residual harmonisation bias. Furthermore, the cross-sectional design identifies associations and group differences but does not permit causal inferences regarding factors influencing CMT-related knowledge and awareness.

Future research should explore physician–patient communication, regulatory awareness and targeted educational strategies to promote the safe and evidence-based integration of CMTs into healthcare practice. Further studies evaluating interventions to enhance knowledge of CMTs among healthcare professionals and the public are warranted. Research on clinical decision-making, patient disclosure of CMT use and communication practices between physicians and patients may also provide valuable insights for strengthening integrative healthcare.

## 5. Conclusions

This exploratory comparative analysis of two independent survey datasets revealed notable differences in CMT-related familiarity, awareness and information-seeking behaviours between family physicians and members of the general population in Saudi Arabia. Family physicians exhibited greater familiarity with CMT concepts and achieved higher overall CMT-related knowledge scores compared to the general public. Nevertheless, both groups demonstrated limited awareness of regulatory frameworks and quality assurance measures, with certain results suggesting slightly higher awareness among public participants than physicians. Therefore, the observed advantage among physicians should be understood as pertaining mainly to specific domains of CMT knowledge that are clearly supported by the data, rather than encompassing all aspects of CMT-related awareness. These results underscore ongoing gaps in understanding of CMT regulation and governance within both populations.

The findings should be interpreted with due consideration of the study’s methodological limitations, including the relatively small physician sample, demographic disparities between the comparison groups and the use of harmonised secondary datasets. Despite these limitations, the results underscore the need to strengthen physician education and continuing professional development in CMTs, particularly in relation to safety, regulation and evidence-informed patient counselling. In addition, targeted public health education strategies may enhance awareness of the safe and appropriate use of CMTs among the general population. Future research should focus on developing validated assessment instruments, examining physician–patient communication regarding CMT use and undertaking larger methodologically robust studies that account for demographic and professional factors influencing CMT-related knowledge and awareness.

## Figures and Tables

**Table 1:** Demographic Characteristics of the Participant Groups of Family Physicians (N = 62) and the Public (N = 1245) in Saudi Arabia.

Variable	Family Physicians, *n* (%)		Public, *n* (%)	
Age (years)	25–35	14 (22.6%)	18–29	700 (56.2%)
	36–45	36 (58.1%)	30–39	222 (17.8%)
	46–55	12 (19.4%)	40–49	204 (16.4%)
	≥56	0 (0.00%)	≥50	119 (9.6%)
Gender	Male	31 (50.0%)	Male	373 (30.0%)
	Female	31 (50.0%)	Female	872 (70.0%)
Education/ professional level	General practitioner	7 (11.3%)	No formal education	0 (0.00%)
	Resident	8 (12.9%)	High school or below	352 (28.3%)
	Specialist	6 (9.7%)	Bachelor’s degree	736 (59.1%)
	Consultant	41 (66.1%)	Postgraduate degree	157 (12.6%)
Monthly income (SAR)	<20,000	7 (11.3%)	<5000	663 (53.3%)
	20,000–<30,000	8 (12.9%)	5000–<10,000	233 (18.7%)
	30,000–40,000	6 (9.7%)	10,000–20,000	230 (18.5%)
	>40,000	41 (66.1%)	>20,000	119 (9.6%)

**Table 2:** Comparative Findings Regarding Knowledge of CMTs among Family Physicians (N = 62) and Members of the Public (N = 1245) in Saudi Arabia.

Knowledge Item	Response	Family Physicians	Public
Conceptual understanding of CMTs	Yes	53 (85.5%)	904 (72.6%)
	No	8 (13.5%)	227 (18.2%)
	Don’t know/Not sure	1 (1.0%)	114 (9.2%)
CMTs regulations and quality control	Yes	13 (21.0%)	306 (24.6%)
	No	20 (32.3%)	533 (42.8%)
	Don’t know/Not sure	29 (46.8%)	406 (32.6%)
CMTs information sources	Healthcare Professionals	41 (66.1%)	433 (34.8%)
	Scientific Online articles	28 (45.2%)	377 (30.3%)
	Friends and Family	14 (22.6%)	305 (24.5%)
	Social media	12 (19.4%)	130 (10.4%)

**Table 3:** Association between Knowledge of CMTs and the Participant Groups: The Public (N = 1245) and Family Physicians (N = 62) in Saudi Arabia.

Knowledge Item	Response	Family Physicians	Public	*p*-Value
Basic knowledge of the CMT concept	Yes	53 (85.5%)	904 (72.6%)	0.020
	No	8 (13.5%)	227 (18.2%)	
	Don’t know/Not sure	1 (1.0%)	114 (9.2%)	
Knowledge of CMT regulations and quality control	Yes	13 (21.0%)	306 (24.6%)	0.050
	No	20 (32.3%)	533 (42.8%)	
	Don’t know/Not sure	29 (46.8%)	406 (32.6%)	
CMT information sources	Healthcare Professionals	41 (66.1%)	433 (34.8%)	<0.001
	Scientific Online articles	28 (45.2%)	377 (30.3%)	
	Friends and Family	14 (22.6%)	305 (24.5%)	
	Social media	12 (19.4%)	130 (10.4%)	
Overall CMT knowledge	Poor	3 (4.8%)	1021 (82.0%)	<0.001
	Average	37 (59.7%)	0 (0.0%)	
	Good	22 (35.5%)	224 (18.0%)	

## Data Availability

The data presented in this study are available on request from the corresponding author due to confidential reason.

## Supplementary Materials

The following supporting information can be downloaded at: https://www.mdpi.com/article/10.3390/healthcare14131930/s1, Table S1. STROBE Checklist Applied to the Comparative Analysis of of the Participant Groups of Family Physicians (N = 62) and the Public (N = 1245) in Saudi Arabia.; Table S2. Complementary and alternative medicine (CAM)—Family Physicians Survey; Table S3. Complementary and alternative medicine (CAM)—Public Survey. Table S4. Harmonisation of Variables Across the Physician and Public Datasets.
