# Supplementary material for: Knowledge of Complementary Medicine and Therapies Among Family Physicians and the General Population in Saudi Arabia
Source: Healthcare (Basel). 2026 Jul 1;14(13):1930. doi: 10.3390/healthcare14131930 (PMC13361361; doi:10.3390/healthcare14131930)
Supplement: Supplementary file 1 [file healthcare-14-01930-s001.zip › healthcare-4369128-supplementary.pdf]

healthcare

MDPI

Table S1. STROBE Checklist Applied to the Comparative Analysis of of the Participant Groups of Family Physicians (N = 62) and the Public (N = 1245) in Saudi Arabia.

STROBE Item	Recommendation	Family Physician Dataset	Public Dataset	Comparative Analysis Manuscript
• Title and Abstract	Indicate study design and summary findings	Cross-sectional study	Cross-sectional study	Comparative cross-sectional secondary analysis
• Background/Rationale	Explain the scientific background and rationale	Assess CAM knowledge, attitudes and practices among family physicians	Assess CAM safety awareness and AI-related perceptions among the public	Compare CMT-related knowledge and information sources between physicians and the public
• Objectives	State-specific objectives	Evaluate family physicians' CAM knowledge	Evaluate public awareness of CAM safety	Compare knowledge, regulatory awareness and information sources
• Study Design	Present key study design elements	Cross-sectional survey	Cross-sectional survey	Comparative secondary analysis of two cross-sectional datasets
• Setting	Describe the setting, location and dates	Saudi Arabia; online national survey	Saudi Arabia; online national survey	Saudi Arabia; analysis of two national datasets
• Participants	Eligibility criteria and recruitment	Family physicians practicing in Saudi Arabia (GPs, residents, specialists and consultants)	Adults ≥18 years residing in Saudi Arabia	Eligible participants with harmonisable variables and complete responses
• Sampling Strategy	Recruitment methods	Online convenience sampling	Online convenience sampling	Secondary analysis of available datasets
• Variables	Define outcomes and explanatory variables	CAM knowledge, attitudes and practices	CAM awareness and AI perceptions	CMT knowledge, regulatory awareness, information sources
• Data Sources/Measurement	Sources of data and assessment methods	Self-administered online questionnaire	Self-administered online questionnaire	Harmonised variables from both datasets
• Bias	Address potential sources of bias	Convenience sampling acknowledged	Convenience sampling acknowledged	Limitations and potential selection bias discussed
• Study Size	Explain sample size	*n* = 62 physicians	n = 1245 public participants	Total *n* = 1307
• Quantitative Variables	Handling of quantitative variables	Knowledge scores categorised as low, average and good	Awareness scores categorised as poor, average and good	Harmonised percentage-based knowledge categories
• Statistical Methods	Statistical analyses used	Chi-square and Fisher's exact tests	Chi-square and Fisher's exact tests	Chi-square and Fisher's exact tests for between-group comparisons
• Missing Data	Describe handling of missing data	Complete responses analysed	Complete responses analysed	Complete-case analysis; no imputation performed
• Participant Flow	Report numbers included and excluded	62 complete responses included	1245 complete responses included	1307 participants included in final analysis

• Descriptive Data	Participant characteristics	Age, gender, professional level, experience	Age, gender, education, occupation, income	Comparative demographic characteristics presented
• Outcome Data	Report outcome measures	CAM knowledge and awareness measures	CAM awareness measures	Comparative knowledge and awareness outcomes
• Main Results	Present estimates and statistical significance	Physician-specific results reported	Public-specific results reported	Significant between-group differences reported ($p < 0.05$)
• Other Analyses	Subgroup or additional analyses	Knowledge vs practice associations	Awareness vs demographic factors	Comparative analysis of harmonised variables
• Key Results	Summarise findings	Moderate-to-good CAM knowledge	Predominantly poor CAM awareness	Physicians demonstrated higher CMT-related knowledge than the public
• Limitations	Discuss study limitations	Cross-sectional design;<br>online recruitment	Cross-sectional design; online recruitment	Secondary data analysis; non-probability sampling; causal inference not possible
• Interpretation	Interpret findings considering evidence	Need for physician education and regulation	Need for public awareness and safety education	Need for targeted educational and regulatory interventions
• Generalisability	Discuss external validity	Limited by online recruitment	Limited by online recruitment	Findings applicable with caution to Saudi Arabian populations
• Ethical Approval	Ethics committee approval	HAPO-02-K-012-2024-032080	HAPO-02-K-012-2025-02-2550	Both approvals reported in Methods
• Funding	Report funding sources	Reported in the original study (no funding)	Reported in the original study (no funding)	Included in manuscript declarations

Table S2. Complementary and alternative medicine (CAM) - Family Physicians Survey.

**Socio-Demographic data**
**Age in years**
• 25–35
• 36–45
• 46–55
**Gender**
• Male
• Female
**Years of experiences**
• < 5 years
• 5–10 years
• > 10 years
**Professional classification**
• General practitioner
• Resident
• Specialist
• Consultant
**Monthly income (SAR)**
• <20,000
• 20,000–<30,000
• 30,000–40,000
• >40,000
**CAM training exposure ***
• Yes
• No
**CAM social media engagement**

- Yes
- No

Knowledge items

Do you know what CAM stands for?
- Yes
- No
- Not sure

Have you ever heard of CAM?
- Yes
- No
- Not sure

How would you rate your knowledge about CAM therapies?
- Limited
- Excellent
- Not sure

Should CAM be an integral part of conventional medicine?
- Yes
- No
- Not sure

What CAM should be?
- Complementary to conventional medicine
- An integral part of conventional medicine
- An alternative to conventional medicine
- Not sure

Should patients be treated exclusively with conventional medicine?
- Yes
- No
- Not sure

Are there clear guidelines about CAM in Saudi Arabia?
- Yes
- No
- Not sure

Source of knowledge about CAM among family physicians

- Healthcare Professionals /healthcare databases
- Scientific Online articles /Books/journals
- Friends / Family
- Internet / Social media
- Others

Table S3. Complementary and alternative medicine (CAM) – Public Survey.

Age (years)
- 18–29
- 30–39
- 40–49
- ≥50

Gender
- Male
- Female

Education /professional level

- No formal education
- High school or below
- Bachelor's degree
- Postgraduate degree

Monthly income (SAR)

- <5000
- 5000–<10,000
- 10,000–20,000
- >20,000

Do you Have a chronic disease ?

- Yes
- No

Type of the disease

- Hypertension
- DM
- High cholesterol level
- Others
- Asthma
- Cardiovascular disease
- Thyroid dysfunction

Smoking

- Yes
- No

What do you understand by the term CAM ?

- A medicine derived entirely from natural sources
- A synthetic medicine with some natural ingredients
- Don't know

Are you aware that CAM can have side effects or interact with other medications?

- Yes
- No
- Don't know

Which of the following safety concerns about CAM do you know about?

- Toxicity of ingredients
- Lack of standard dosages
- Contamination with harmful substances
- Interactions with conventional medicines

Do you know of any regulatory measures for CAM in your country?

- Yes
- No
- Don't know

Do you believe CAM undergo the same safety scrutiny as conventional drugs?

- Yes
- No
- Don’t know

Are you currently using CAM?

- Yes
- No
- Don’t know

Frequency of using CAM

- Daily
- Weekly
- Monthly
- Annually

How often do you consider the safety of CAM before using them?

- Never
- Rarely
- Occasionally
- Often

When choosing CAM , what factors do you consider?

- Recommendations from friends or family
- Advice from a healthcare professional
- Online Scientific reviews
- Social media or Brand reputation
- No specific considerations

What steps do you take to ensure the safety of the CAM you use?

- Check the product's certificates or labels
- Search for the manufacturer
- Consult a healthcare professional
- No specific actions

Experienced side effects from using CAM

- Yes
- No
- Don’t know

Reported an adverse effect of CAM s to any regulatory body or platform

- No Yes
- No
- Don’t know

Heard about the use of artificial intelligence in monitoring or improving drug safety, including herbal remedies

- Yes
- No
- Don't know

What do you understand about the role of artificial intelligence in healthcare?

- Artificial intelligence can help with drug safety
- Artificial intelligence can diagnose diseases
- Artificial intelligence has no role in healthcare

Have you come across any educational materials or campaigns that address the role of artificial intelligence in the safety of CAM ?

- Yes
- No
- Don't know

Which of the following applications of AI in CAM safety are you familiar with?

- Detecting toxic substances in herbal medications
- Monitoring adverse effects of herbal products
- Analyzing drug-herb interactions
- I am not aware of any applications

Table S4. Harmonisation of Variables Across the Physician and Public Datasets.

Analytical Variable	Original Physician Dataset	Original Public Dataset	Conceptual Equivalence Assessment	Original Response Structure	Harmonisation Procedure	Final Analytical Categories	Retained in Comparative Analysis	Justification
• Study Group	Family physicians	General public	Complete equivalence	Physician / Public	Direct coding	Physician / Public	Yes	Primary exposure variable
• Gender	Male, Female	Male, Female	Complete equivalence	Binary	No recoding required	Male / Female	Yes	Identical construct and response options
• Knowledge Level	Overall CAM knowledge score (Poor, Average, Good)	Overall CAM knowledge score (Poor, Average, Good)	Partial equivalence	Different scoring structures	Direct coding	Poor, Average, Good	Yes	Allowed comparison of overall knowledge status across both datasets
• Regulatory Awareness	Are there clear CAM guidelines in Saudi Arabia	Do you know of any regulations or quality-control measures for CAM ?	High conceptual equivalence	Yes / No / Not Sure	Not Sure and Don't Know combined	Yes<br>No<br>Don't know / Not sure	Yes	Both variables assessed awareness of regulatory oversight

Analytical Variable	Original Physician Dataset	Original Public Dataset	Conceptual Equivalence Assessment	Original Response Structure	Harmonisation Procedure	Final Analytical Categories	Retained in Comparative Analysis	Justification
• Professional Information Sources	Peer-reviewed journals, professional resources	Healthcare professionals	Moderate conceptual equivalence		Grouped as professional healthcare information sources	Yes / No	Yes	Both reflect professionally sourced health information
• Online Information Sources	Internet	Online articles/blogs	High conceptual equivalence	Multiple response items	Combined into online information category	Yes / No	Yes	Both represent web-based information seeking
• Family/Friends Information Source	Family/Friends	Family/Friends	Complete equivalence	Multiple response items	Direct harmonisation	Yes / No	Yes	Identical information source category
• Age	25–35, 36–45, 46–55 years	18–29, 30–39, 40–49, ≥50 years	Limited equivalence	Different category boundaries	Descriptive	Partially applicable	Yes	Categories were not directly comparable
• Education	Professional classification (GP, Resident, Specialist, Consultant)	Educational attainment (High School, Bachelor's, Postgraduate)	Limited equivalence	Different category boundaries	Descriptive	Partially applicable	Yes	Education /professional level is not directly comparable
• Income	Physician income categories	Public income categories	Limited equivalence	Different category boundaries	Descriptive	Partially applicable	Yes	Categories were not directly comparable
• Attitudes Toward CAM	Multiple Likert-scale attitude items	Not collected	No equivalence	Different measurement structure	Excluded	Not applicable	No	No comparable variable available
• Clinical Practice	Recommendation of CAM therapies	CAM use behaviour	No equivalence	Different behavioural constructs	Excluded	Not applicable	No	Physician practice cannot be directly compared with public use behaviour
• AI Awareness	Not collected	AI awareness and perceptions	No equivalence	Variable absent in physician dataset	Excluded	Not applicable	No	Variable unavailable in one dataset
• CAM Counselling Confidence	Collected in physicians	Not collected	No equivalence	Variable absent in public dataset	Excluded	Not applicable	No	Variable unavailable in one dataset
• Beliefs Regarding CAM Effectiveness	Collected in physicians	Not collected	No equivalence	Variable absent in public dataset	Excluded	Not applicable	No	Variable unavailable in one dataset
